# Comparison of the efficacy and safety of TACE-HAIC-MTTs-ICIs and TACE-MTTs-ICIs in the hepatocellular carcinoma: a prognostic analysis based on the dynamic changes of serum AFP

**DOI:** 10.1097/JS9.0000000000002818

**Published:** 2025-06-20

**Authors:** Wenli Li, Mingjian Lu, Guosheng Yuan, Feng Shi, Mengya Zang, Xiaoyun Hu, Qi Li, Peilin Zhu, Yongru Chen, Jingyi Zhao, Kaiyan Su, Yunheng Peng, Jinzhang Chen

**Affiliations:** a Department of Infectious Diseases, Nanfang Hospital, Guangdong Provincial Key Laboratory for Prevention and Control of Major Liver Diseases, Southern Medical University, Guangzhou, China; b Department of Interventional Radiology, Guangzhou Institute of Cancer Research, Affiliated Cancer Hospital, Guangzhou Medical University, Guangzhou, China; c Department of Interventional Radiology, Guangdong Provincial People’s Hospital, Guangdong Academy of Medical Sciences, Southern Medical University, Guangzhou, China; d Department of Hepatobiliary Surgery, Shantou Central Hospital, Shantou, China

**Keywords:** AFP trajectory, HAIC, hepatocellular carcinoma, ICI, MTT, survival, TACE

## Abstract

**Background:**

To evaluate the efficacy and safety of two combination treatments for hepatocellular carcinoma (HCC): 1) transarterial chemoembolization (TACE) with hepatic arterial infusion chemotherapy (HAIC) combined with molecular targeted therapies (MTTs) and immune checkpoint inhibitors (ICIs) (TACE-HAIC-MI), and 2) TACE combined with MTTs and ICIs (TACE-MI). In addition, analysis of changes in serum AFP levels and patient survival was performed.

**Methods:**

A retrospective study of 459 HCC patients treated with one of the aforementioned treatments was performed: TACE-MI (*N* = 305) and TACE-HAIC-MI (*N* = 154). Inverse probability of treatment weighting (IPTW) was used to minimize confounding factors, and sensitivity analyses were conducted. The joint latent class model was used to model AFP change trajectories, and Cox regression analysis was used to identify prognostic factors. Primary and secondary endpoints were overall survival (OS) and progression-free survival (PFS), respectively. Tumor response and adverse events (AEs) were assessed using RECIST v1.1 and CTCAE v5.0, respectively.

**Results:**

After IPTW, the median OS of the TACE-HAIC-MI group was 23.9 months, and for the TACE-MI group it was 21.7 months(*P* = 0.432). The median PFS of the two groups was 9.77 months versus 8.97 months (*P* = 0.45). Six optimal latent classes of AFP trajectories were identified. Class 1 demonstrated a median OS of 20.43 months, while class 2 did not reach the median OS. The median OS for class 3 was 43.87 months, for class 4 was 18.47 months, for class 5 was 8.13 months, and for class 6 was 11.37 months. Cox regression analyses showed that AFP trajectory independently predicted median OS and PFS, with HRs between 1.56 and 45.28. The most common AEs were elevated transaminase levels, electrolyte imbalances, anemia, and pain. Nausea and vomiting were more frequent in the TACE-HAIC-MI group, and elevated transaminase levels were less common than in the TACE-MI group.

**Conclusion:**

TACE-HAIC-MI and TACE-MI treatments have no significant survival differences in patients with HCC. Patients with a favorable AFP response have significantly better survival outcomes than those with a poor response.

## Introduction

Despite advances in early diagnosis, hepatocellular carcinoma (HCC) remains a leading cause of cancer-related mortality globally^[1,2]^. The combination of immune checkpoint inhibitors (ICIs) and molecular targeted therapies (MTTs) has improved outcomes for patients with advanced HCC, but the median overall survival (OS) is still <2 years and thus new treatments are needed^[3]^.

Recently, the combination of interventional therapies and systemic treatments has been shown to be effective against HCC with notable survival benefits^[4,5]^. Studies have shown that transarterial chemoembolization (TACE) combined with MTTs and ICIs (referred to as TACE-MI) outperforms single local or systemic treatments in terms of tumor response and survival with manageable safety profiles^[6-8]^. Other studies have indicated that combining hepatic arterial infusion chemotherapy (HAIC) with MTTs and ICIs may extend the survival of patients with HCC^[9]^. TACE causes tumor ischemic necrosis by blocking the blood supply, releasing antigens, and activating the immune system^[5]^. Targeted therapy inhibits angiogenesis and improves the tumor microenvironment^[10]^. HAIC boosts the local drug concentration to attack tumor cells, whereas immunotherapy activates T cells to enhance the immune response. Theoretically, quadruple therapy of TACE with HAIC plus MTTs, and ICIs (known as TACE-HAIC-MI) would be expected to achieve better outcomes. However, combination therapies are currently under investigation.

Alpha-fetoprotein (AFP) is an important biomarker for diagnosing and predicting HCC outcomes; however, its sensitivity and specificity are limited^[11]^. Numerous studies have shown that elevated AFP levels are closely related to poor prognosis and can predict treatment outcomes across various disease stages^[12–14]^. Some studies have reported that AFP combined with other markers, such as des-gamma-carboxy prothrombin may improve prognostic accuracy^[15]^. The challenge lies in the inconsistent AFP changes across different stages and treatments. Recent studies have highlighted different classification criteria affecting prognosis^[16,17]^. Dynamic AFP levels have been shown to improve prognostic accuracy in patients with HCC treated with bevacizumab and immunotherapy^[18]^. To our knowledge, no study has examined the relation between dynamic AFP change patterns and survival prognosis in the context of treatment with TACE with or without HAIC plus MTTs and ICIs. Evaluating overall dynamic AFP changes could fill current gaps and enhance the management of HCC.

Thus, the purpose of this study was to compare the effectiveness and safety of TACE-HAIC-MI and TACE-MI treatments for HCC and analyze the association between dynamic AFP changes and survival outcomes. The special declaration was that all artificial intelligence (AI) applications in this research comply with transparency standards outlined in the TITAN 2025 Guidelines^[19]^.

## Materials and methods

### Included patients

In this retrospective multicenter study, we analyzed 616 HCC patients treated with combination intervention and systemic therapy from January 2019 to December 2023. A total of 459 patients met the inclusion criteria and were included in the analysis.

**Figure 1.**
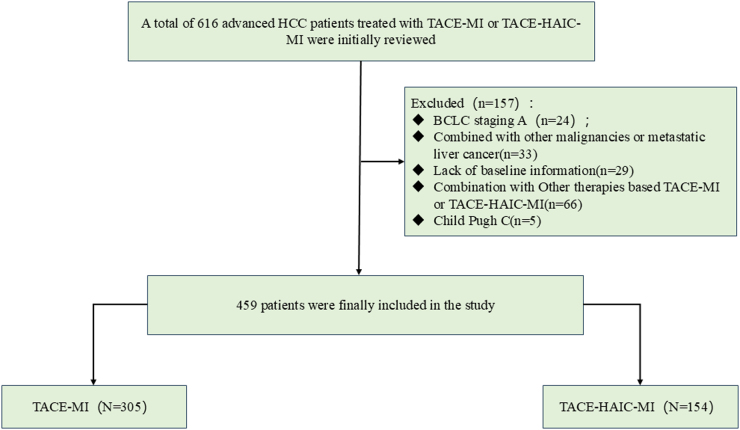
The flowchart of patients. Abbreviations: TACE-MI, transarterial chemoembolization (TACE) combined with molecular targeted therapies (MTTs) and immune checkpoint inhibitors (ICIs); TACE-HAIC-MI, TACE with hepatic arterial infusion chemotherapy (HAIC) combined with MTTs and ICIs; BCLC, Barcelona Clinic Liver Cance.

A flow diagram of patient inclusion and exclusion is shown in Figure 1. The inclusion criteria were as follows: (1) Clinical or pathological diagnosis of HCC, (2) Age greater than 18 years, (3) Liver function classified as Child-Pugh A5 or B8, (4) Availability of relatively complete baseline and dynamic follow-up data for AFP, and (5) ECOG performance status (PS) score of 0–1.

The exclusion criteria were as follows: (1) Barcelona Clinic Liver Cancer (BCLC) stage A HCC; (2) Patients with coexisting malignancies or radiologically/histologically proven metastatic liver cancer; (3) Missing data of interest; (4) Received other treatments than those being examined in this study; and (5) Child-Pugh B9 or C liver function.

The Ethics Committee of our hospital (details omitted for double-anonymized peer review) has approved this study in accordance with the STROCCS guidelines^[20]^.

**HIGHLIGHTS**

- There were no significant differences in overall survival or progression-free survival in patients with HCC treated with TACE-MI and TACE-HAIC-MI.
- Six dynamic AFP change trajectories were identified using JLCM. Patients with persistently low levels of AFP in class 2 showed the longest median OS. Class 3, characterized by a high baseline AFP level and a persistent decline approaching normal levels after treatment, had the longest OS of 43.87 months. Patients with a poor AFP response, characterized by persistent elevation of AFP levels after treatment, have an extremely poor prognosis.
- TACE-HAIC-MI was associated with higher nausea and vomiting rates, but lower elevated transaminase levels than the TACE-MI group. Both groups had a 51% to 84% incidence of adverse reactions with no treatment-related deaths, indicating acceptable safety profiles.

### Data collection

Patient data was extracted from medical records across four research centers using the same protocol and included: sex, age, ECOG performance status, the presence of radiologic cirrhosis, liver function classification, maximum tumor diameter, number of tumors, vascular invasion, extrahepatic metastasis, BCLC stage, AFP levels, blood hematological and biochemical indices, type of treatment, and adverse events (AEs). The follow-up date for all patients was censored on 30 November 2024. The OS was measured from the start of treatment to death or the last follow-up. Progression-free survival (PFS) was measured from the start of treatment to disease progression or death. Two oncologists independently assessed the tumor response and resolved any differences through discussions.

## Treatments

### TACE

TACE included conventional TACE (cTACE) and drug-eluting bead TACE (DEB-TACE) and was performed using super-selective catheterization and embolization techniques, with a primary focus on preserving maximal liver function. The selection and dosage of chemotherapeutic and embolic agents used during TACE sessions adhered to the guideline of Chinese Clinical Practice Guidelines for the Diagnosis and Treatment of Primary Liver Cancer. In accordance with guidelines, repeat TACE was considered in cases of significant viable tumors or intrahepatic recurrence during follow-up imaging assessments.

### HAIC

For the HAIC procedure, a catheter or microcatheter was positioned within the primary feeding hepatic artery under the guidance of digital subtraction angiography following femoral artery puncture using the Seldinger technique. The chemotherapeutic regimen was administered via the hepatic artery. The FOLFOX regimen comprised oxaliplatin at a dosage of 85 mg/m^2^ administered from hours 0 to 4 on day 1, leucovorin at 400 mg/m^2^ from hours 4 to 5 on day 1, and a 5-fluorouracil bolus of 400 mg/m^2^ at hour 5, followed by a continuous infusion of 2400 mg/m^2^ over 46 h on days 1 and 2. The RALOX protocol was oxaliplatin at 100 mg/m^2^ from hour 0 to 4 on day 1, and raltitrexed at 3 mg/m^2^ from hour 4 to 5 on day 1.

### Definition of combined therapy

TACE combined with HAIC therapy involves two treatment modes. For Sequential Mode, a catheter is left in the tumor-feeding artery after TACE for continuous chemotherapy infusion. For Phased Mode, HAIC is administered 3-4 weeks after TACE. Targeted therapy and ICIs can be administered 1 month before or up to 3 months after interventional therapy.

### AFP measurement

AFP quantification was performed by medical professionals using electrochemiluminescence immunoassays at all participating centers. Two chemiluminescence platforms were used: Roche Cobas E601 and Mindray CL-6000i analyzers. All centers adhere to a uniform normal reference range (0–7 ng/mL). However, there were differences in upper detection limits (54 000 ng/mL, 121 000 ng/mL, and 187 500 ng/mL). Values beyond these limits were recorded as maximum values. The baseline AFP level was measured within 2 weeks before treatment, and then levels were measured every about 4–8 weeks post-treatment.

### *Inverse probability of treatment weighting analysis* ^[21]^

Inverse probability of treatment weighting (IPTW) analysis is comprised of the following steps. 1) Data Preparation: All baseline characteristics were included (Table 1). 2) Propensity Score Estimation: A logistic regression model was developed to estimate propensity scores, with treatment group (TACE-MI vs. TACE-HAIC-MI). 3) Stabilized inverse probability weights were calculated for each patient based on their estimated propensity scores, with separate weight definitions for the treatment (TACE-HAIC-MI) and control (TACE-MI) groups. 4) Weights were applied to generate a balanced pseudopopulation. 5) Standardized mean differences (SMD) were evaluated before and after weighing. Covariate balance was visualized with SMD in supplementary Figure S1. http://links.lww.com/JS9/E459. Sensitivity analyses included: a comparison of unweighted and weighted effect estimates with different weighting methods.

**Table 1:** Baseline characteristics of TACE-HAIC-MI and TACE-MI group

Characteristics		Unmatched				After IPTW			
	Group	TACH-HAIC-MI	TACE-MI	*P*	SMD	TACH-HAIC-MI	TACE-MI	*P*	SMD
	*N*	305	154			456	454		
Sex (%)	Female	36 (11.8)	14 (9.1)	0.47	0.089	51 (11.2)	42 (9.2)	0.56	0.065
	Male	269 (88.2)	140 (90.9)			405 (88.8)	412 (90.8)		
Age (%)	<60	204 (66.9)	112 (72.7)	0.242	0.128	312 (68.4)	315 (69.4)	0.863	0.021
	≥60	101 (33.1)	42 (27.3)			144 (31.6)	139 (30.6)		
ECOG PS (%)	0	233 (76.4)	109 (70.8)	0.234	0.128	340(74.5)	344 (75.8)	0.777	0.031
	1	72 (23.6)	45 (29.2)			116 (25.5)	110 (24.2)		
Etiology (%)	Other	35 (11.5)	21 (13.6)	0.605	0.065	54 (11.8)	56 (12.3)	0.907	0.014
	Virus	270 (88.5)	133 (86.4)			402(88.2)	398 (87.7)		
Vascular.invasion (%)	Absent	177 (58.0)	51 (33.1)	<0.001	0.517	230 (50.4)	234 (51.5)	0.852	0.022
	Present	128 (42.0)	103 (66.9)			226 (49.6)	220 (48.5)		
Metastasis (%)	Absent	210 (68.9)	81 (52.6)	0.001	0.338	287 (62.9)	271 (59.7)	0.575	0.066
	Present	95 (31.1)	73 (47.4)			169 (37.1)	183.0 (40.3)		
BCLC (%)	B	136 (44.6)	29 (18.8)	<0.001	0.576	164 (36.0)	154 (33.9)	0.728	0.046
	C	169 (55.4)	125 (81.2)			292 (64.0)	300 (66.1)		
Tumor.number (%)	>3	176 (57.7)	100 (64.9)	0.164	0.149	275 (60.3)	267 (58.8)	0.798	0.032
	≤3	129 (42.3)	54 (35.1)			181 (39.7)	187 (41.2)		
ALBI (%)	1	111 (36.4)	29 (18.8)	<0.001	0.401	139 (30.5)	126 (27.8)	0.606	0.065
	2 or 3	194 (63.6)	125 (81.2)			317 (69.5)	328 (72.2)		
AFP (%)	>400	128 (42.0)	89 (57.8)	0.002	0.321	212 (46.5)	204 (44.9)	0.778	0.033
	≤400	177 (58.0)	65 (42.2)			244 (53.5)	250 (55.1)		
Largest.tumor (%)	>10	87 (28.5)	78 (50.6)	<0.001	0.464	164 (36.0)	167 (36.8)	0.883	0.017
	≤10	218 (71.5)	76 (49.4)			292 (64.0)	287 (63.2)		
Imaging.cirrhosis (%)	Absent	138 (45.2)	85 (55.2)	0.056	0.2	221 (48.5)	217 (47.8)	0.897	0.016
	Present	167 (54.8)	69 (44.8)			235 (51.5)	237 (52.2)		
ALT (%)	>40	132 (43.3)	90 (58.4)	0.003	0.307	219 (48.0)	210 (46.3)	0.766	0.035
	≤40	173 (56.7)	64 (41.6)			237 (52.0)	244 (53.7)		
AST (%)	>40	177 (58.0)	122 (79.2)	<0.001	0.469	296 (65.0)	285 (62.9)	0.738	0.044
	≤40	128 (42.0)	32 (20.8)			160 (35.0)	169 (37.1)		
PLT (%)	>100	255 (83.6)	144 (93.5)	0.005	0.315	395 (86.6)	390 (85.9)	0.89	0.021
	≤100	50 (16.4)	10 (6.5)			61 (13.4)	64 (14.1)		
PT (%)	>13.5	35 (11.5)	28 (18.2)	0.068	0.19	57 (12.5)	56 (12.3)	0.966	0.004
	≤13.5	270 (88.5)	126 (81.8)			399 (87.5)	398 (87.7)		
History (%)	No	237 (77.7)	146 (94.8)	<0.001	0.513	381 (83.6)	401 (88.3)	0.34	0.143
	Yes	68 (22.3)	8 (5.2)			75 (16.4)	53 (11.7)		

AFP, Alpha-Fetoprotein; ALBI, Albumin-Bilirubin; ALT, Alanine Aminotransferase; AST, Aspartate Aminotransferase; BCLC, Barcelona Clinic Liver Cancer; ECOG PS, Eastern Cooperative Oncology Group Performance Status; IPTW, Inverse probability of treatment weighting; PLT, Platelet Count; PT, Prothrombin Time.

### Modeling methods

The **Joint Latent Class Model (JLCM)**^[22]^ was used to jointly model longitudinal and survival data. Considering the skewed distribution of serum AFP, we applied a logarithmic transformation to AFP. The specific modeling steps are as follows.

### Model construction

Initially, 1 trajectory class was used, and the number gradually increased to 5, as suggested by previous studies. Using Bayesian Information Criterion (BIC), we found five classes to be optimal. However, this configuration only converged to a local maximum rather than the global maximum. Therefore, we subsequently expanded the analysis to seven trajectory classes to achieve better model convergence. To address the local maxima in the mixed modeling, we employed a grid search with 10 replicates and 15 iterations to select the best model.

### Longitudinal model

Given the varying follow-up times and AFP monitoring intervals for each patient, the dynamic changes were modeled using log-transformed AFP values with a quadratic polynomial time term to capture the nonlinear changes over time. The treatment regimen was included as a covariate to assess the impact of the different treatment regimens on AFP levels. The random effects component also included a quadratic polynomial time term to account for individual differences.

### Survival model

Survival data were modeled using a Weibull proportional hazards model, with treatment as a covariate, to evaluate the impact of different treatment regimens on survival.

### Parameter estimation

The model parameters were estimated using the Maximum Likelihood Estimation (MLE) method. The jointlcmm function fits the joint model using maximum likelihood estimation (MLE) and calculates the log-likelihood, along with information criteria including the Bayesian Information Criterion (BIC), Akaike Information Criterion (AIC), Sample-Size Adjusted Bayesian Information Criterion (SABIC), as well as entropy. Finally, the final model was determined by comparing the goodness-of-fit based on various parameters (BIC, Entropy, AIC, SABIC) and integrating clinical experience.

### Outcomes

The primary endpoint was OS, defined as the time from treatment initiation to cancer-related death or last follow-up. The secondary endpoint was PFS, defined as the time from treatment start to disease progression, death, or last follow-up. Tumor response was assessed using RECIST version 1.1. Dynamic AFP trajectories were identified using the JLCM, and OS and PFS curves were plotted using the Kaplan–Meier method.

## Data analysis

The Mann-Whitney U test was used to compare continuous variables between treatment groups, and Fisher’s exact test was used to compare categorical variables. Survival analysis was performed using the Kaplan–Meier method and the log-rank test. AFP trajectories were constructed using the R package lcmm (v2.1.0). To address unmeasured confounding, IPTW with marginal structural models was used to balance the baseline characteristics before survival analysis. Statistical significance was set at *P* < 0.05. Statistical analyses were performed using SPSS version 25.0, software (SPSS, Inc., Chicago, IL, USA) and R version 4.4.2 software (R Foundation, Vienna, Austria).

Postauthoring text refinement was performed using the DeepSeek-R1 web interface (https://chat.deepseek.com). The AI tool exclusively assisted with:

- Grammar and syntax correction
- Academic terminology standardization
- Sentence structure optimization

We confirm that no confidential or sensitive data was disclosed during the AI-assisted revision process. All AI-suggested edits underwent dual human verification (the first and corresponding authors), and the intellectual content and integrity of the manuscript remain the responsibility of the authors.

## Results

1. **Patient Baseline Characteristics**

A total of 459 patients with HCC with a median follow-up time of 29.9 months (95% CI: 27.5–32.3 months) were included in the analysis. There were 305 patients in the TACE-MI group and 154 patients in the TACE-HAIC-MI group. Initially, the groups differed in terms of vascular invasion, BCLC stage, ALBI grade, tumor size, aspartate aminotransferase (AST) level, and treatment history. IPTW analysis revealed that these differences were no longer present (Table 1). The median number of treatments varied; however, the proportion of patients who received ≤4 treatments was similar. There were no differences between the MTTs, and ICIs used (Supplementary material, Table S1. http://links.lww.com/JS9/E459).

1. **Efficacy and Tumor Response Analysis**

**Figure 2.**
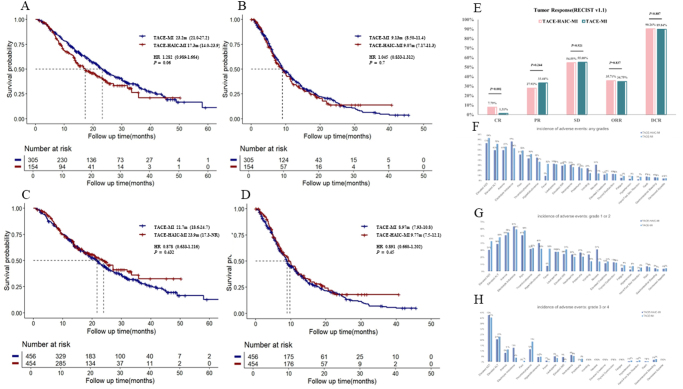
Analysis of survival, tumor response, and adverse events. OS(A) and PFS(B) between TACE-HAIC-MI and TACE-MI group before IPTW. OS(C) and PFS(D) between TACE-HAIC-MI and TACE-MI group after IPTW analysis. Tumor response of TACE-HAIC-MI and TACE-MI group(E). Incidence of adverse events for any grades (F), grade 1 or 2(G), and grade 3 or 4(H) between TACE-HAIC-MI and TACE-MI group. Abbreviations: OS, overall survival; PFS, progression-free survival; TACE-MI, transarterial chemoembolization (TACE) combined with molecular targeted therapies (MTTs) and immune checkpoint inhibitors (ICIs); TACE-HAIC-MI, TACE with hepatic arterial infusion chemotherapy (HAIC) combined with MTTs and ICIs. IPTW, inverse probability of treatment weighting. CR, complete response; PR, partial response; SD, stable disease; ORR, overall response rate; DCR, disease control rate.

Before IPTW, the median OS in the TACE-MI group was 23.2 months (95% CI: 21.0–27.2 months), compared to 17.3 months (95% CI: 14.0–23.9 months) in the TACE-HAIC-MI group (*P* = 0.06). The median PFS was 9.13 months (95% CI: 8.5–11.4 months) in the TACE-MI group and 9.07 months (95% CI: 7.17–11.3 months) in the TACE-HAIC-MI group (*P* = 0.7) (Fig. 2A and 2B). After IPTW, the median OS was 21.7 months (95% CI: 18.6–21.7 months) in the TACE-MI group and 23.9 months (95% CI, 17.3–NA months) in the TACE-HAIC-MI group (*P* = 0.432). The median PFS was 8.97 months (95% CI: 7.93–10.8 months in the TACE-MI group and 9.77 months (95% CI: 7.5–12.1 months) in the TACE-HAIC-MI group (*P* = 0.45) (Fig. 2C and 2D). Similar results were obtained using different weighting methods (Supplementary Material Figure 1. http://links.lww.com/JS9/E459).

In the TACE-HAIC-MI cohort, 12 patients (7.79%) achieved a complete response, in contrast to 4 patients (1.31%) in the TACE-MI cohort, with statistical significance (*P* = 0.001). The ORR in the TACE-HAIC-MI and TACE-MI groups were 35.71% versus 34.75%, respectively (*P* = 0.837), demonstrating no statistically significant difference between the two treatment arms. Similarly, the DCR was 90.26% versus 89.84% in the respective groups (*P* = 0.887), indicating comparable efficacy in terms of disease stabilization (Fig. 2E).

1. **Safety Analysis**

The TACE-MI group had a higher frequency of AST elevation (84% vs. 73%, *P* = 0.013), ALT elevation (72% vs. 60%, *P* = 0.011), fever (32% vs. 9%, *P* < 0.001), and hypertension (9% vs. 3%, *P* = 0.031) compared to the TACE-HAIC-MI group. Conversely, the TACE-HAIC-MI group had a higher frequency of nausea (31% vs. 14%, *P* < 0.001) and vomiting (24% vs. 14%, *P* = 0.014), both of which were mild. Other AEs were similar between the two groups. With respect to Grade 1 or 2 AEs, the TACE-MI group had a higher frequency of AST elevation (43% vs. 31%, *P* = 0.01), fever (32% vs. 8%, *P* < 0.001), and hypertension (8% vs. 3%, *P* = 0.04). For Grade 3 or higher AEs, the TACE-HAIC-MI group had higher frequencies of electrolyte imbalances (13% vs. 4%, *P* < 0.001) and international normalized ratio (INR) elevation (5% vs. 0%, *P* < 0.001). Severe proteinuria was more frequent in the TACE-MI group (3% vs. 0%, *P* = 0.03). No treatment-related deaths occurred (Fig. 2F, 2G, 2H, Table S5. http://links.lww.com/JS9/E459).

1. **AFP Trajectory Modeling**

**Figure 3.**
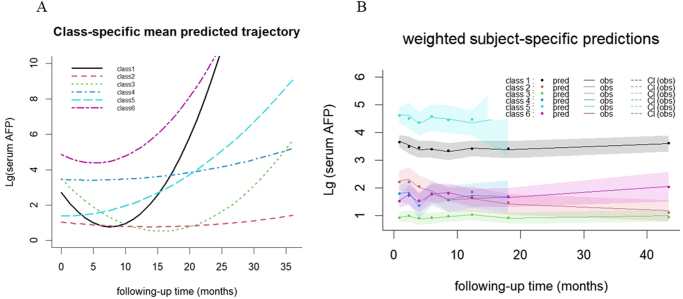
Class-specific predicted trajectory and weighted predictions.

As shown in Supplementary Table 2. http://links.lww.com/JS9/E459, models mj2 and mj2a exhibited a relatively balanced distribution across the 2 classes, with class 1 comprising 60.51% and class 2 comprising 39.49%. Subsequently, models mj3 and mj3a depicted class distributions with mj3 showing 48.13%, 23.60%, and 28.27% for classes 1, 2, and 3, respectively. Models mj4–mj7 further increased the number of classes, resulting in a more intricate distribution pattern. Notably, the mj6 model exhibited a 0% distribution for class 1, suggesting that the model did not converge to a global maximum value. Therefore, it adopts a model with a grid search that is superior in terms of parameters compared with models with the same number of classes. Conversely, model mj6a demonstrated a relatively even distribution across the seven classes: class 1, 10.98%; class 2, 35.98%; class 3, 6.54%; class 4, 17.52%; class 5, 16.12%; and class 6, 12.85%. Further details regarding the model parameters are provided in Supplementary Tables S3 and S4. Supplementary Figure S2. http://links.lww.com/JS9/E459 shows that the mj6a model had the highest BIC values, outperforming AIC, entropy, and SABIC. Model mj6a identified in total six different latent classes for AFP. Figure 3A displays the mean predicted trajectories of dynamic serum AFP levels across six defined subject subgroups, revealing distinct AFP change patterns among subgroups. Figure 3B demonstrates calibrated predictive accuracy by comparing individual predicted trajectories with actual observations in those subgroups.

1. **Association Between AFP Trajectories and Clinical Outcomes**

**Figure 4.**
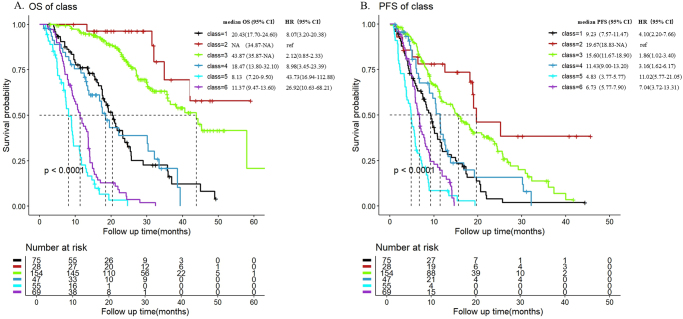
Analysis of OS(A) and PFS(B) for different classes in mj6a model. Abbreviations: OS, overall survival; PFS, progression-free survival.

As shown in Figure 4, there were significant differences in the mean predicted AFP trajectories for each class. The median OS and PFS for each trajectory subgroup were calculated. The median OS was 20.43 months (95% CI: 17.7–24.6 months) for class 1, not reached for class 2 with a lower limit of the 95% CI at 34.87 months, 43.87 months (95% CI: 35.87–NA months) for class 3, 18.47 months (95% CI: 13.8–32.1 months) for class 4, 8.13 months (95% CI: 7.2–9.5 months) for class 5, and 11.37 months (95% CI: 9.47–13.6 months) for class 6 (*P* < 0.0001). The median PFS for classes 1 to 6 were 9.23 months (95% CI: 7.57–11.47 months), 19.67 months (95% CI: 18.83–NA months), 15.60 months (95% CI: 11.67–18.9 months), 11.43 months (95% CI: 9.00–13.20 months), 4.83 months (95% CI: 3.77–5.77 months), and 6.73 months (95% CI: 5.77–7.90 months), respectively (*P* < 0.0001).

Patients in class 2, which was characterized by persistently low AFP levels, had the best prognosis with a median OS that was not reached and was expected to exceed 50 months. Patients with high baseline AFP levels who showed a good response to treatment, with AFP levels decreasing to near-normal values (class 3), had a median OS of 43.87 months. Even patients with extremely high baseline AFP levels (>10 000 ng/ml, class 6) who exhibited a downward trend in AFP after treatment, albeit not substantial, had a higher median OS than those with lower baseline AFP levels, but a continuously increasing trend (class 5).

1. **Cox Regression Analysis and Subgroup Analysis**

Univariate and multivariate Cox regression analyses revealed that AFP trajectory was an independent predictive factor for both median OS and PFS (Tables 2 and 3). In the analysis of dynamic AFP categories regarding influencing factors for OS, the hazard ratios (HR) for classes 1, 3, 4, 5, and 6, relative to class 2 were 8.14 (95% CI: 3.19-20.80, *P*<0.0011.82 (95% CI: 0.65-5.05, *P* = 0.252), 7.46 (95% CI: 2.76-20.19, *P*<0.001), 45.28 (95% CI: 17.23-119.03, *P*<0.001), and 24.17 (95% CI: 8.43-69.27, *P*<0.001), respectively. Regarding influencing factors for PFS, the HRs for classes 1, 3, 4, 5, and 6, as opposed to class 2, were 4.18 (95% CI: 2.19-7.99, *P*<0.001), 1.56 (95% CI: 0.75-3.25, *P* = 0.231), 2.73 (95% CI: 1.34-5.57, *P* = 0.006), 6.03 (95% CI: 2.80-12.98, *P*<0.001), and 12.65 (95% CI: 6.49-24.66, *P*<0.001).

**Table 2:** Univariate and multivariate analysis of influencing factors for PFS (Cox regression)

Characteristic	Univariable					Multivariable				
	*N*	Event *N*	HR^a^	95% CI^a^	*P*-value	*N*	Event *N*	HR^a^	95% CI^a^	*P*-value
ECOG										
0	318	239	—	—		318	239	—	—	
1	110	82	1.40	1.09, 1.81	0.008	110	82	1.37	1.05, 1.80	0.020
BCLC										
B	158	112	—	—		158	112	—	—	
C	270	209	1.37	1.09, 1.72	0.008	270	209	1.25	0.81, 1.94	0.307
Vascular invasion										
Absent	214	159	—	—		214	159	—	—	
Present	214	162	1.12	0.90, 1.39	0.319	214	162	0.64	0.44, 0.92	0.017
Tumor number										
≤3	174	129	—	—		174	129	—	—	
>3	254	192	1.15	0.92, 1.44	0.218	254	192	1.08	0.85, 1.38	0.519
Largest tumor(cm)										
≤10	275	205	—	—		275	205	—	—	
>10	153	116	1.34	1.07, 1.69	0.011	153	116	0.94	0.70, 1.26	0.697
Metastasis										
Absent	277	199	—	—		277	199	—	—	
Present	151	122	1.60	1.27, 2.00	<0.001	151	122	1.43	1.05, 1.96	0.025
Imaging cirrhosis										
Absent	213	160	—	—		213	160	—	—	
Present	215	161	0.99	0.79, 1.23	0.925	215	161	1.05	0.82, 1.34	0.725
ALT(U/L)										
≤40	221	163	—	—		221	163	—	—	
>40	207	158	1.07	0.86, 1.33	0.573	207	158	0.97	0.74, 1.27	0.817
AST(U/L)										
≤40	154	113	—	—		154	113	—	—	
>40	274	208	1.34	1.06, 1.69	0.013	274	208	0.98	0.70, 1.38	0.904
ALBI										
1	134	99	—	—		134	99	—	—	
2 or 3	294	222	1.09	0.86, 1.38	0.493	294	222	1.05	0.80, 1.39	0.719
AFP (ng/ml)										
≤400	228	165	—	—		228	165	—	—	
>400	200	156	1.25	1.01, 1.56	0.044	200	156	0.83	0.54, 1.27	0.393
PLT(10*^9^/L)										
≤100	52	40	—	—		52	40	—	—	
>100	376	281	1.00	0.71, 1.39	0.981	376	281	0.89	0.61, 1.30	0.555
Class										
2	28	12	—	—		28	12	—	—	
1	75	63	4.10	2.20, 7.66	<0.001	75	63	4.18	2.19, 7.99	<0.001
3	154	99	1.86	1.02, 3.40	0.044	154	99	1.56	0.75, 3.25	0.231
4	47	33	3.16	1.62, 6.17	<0.001	47	33	2.73	1.34, 5.57	0.006
6	69	62	7.04	3.72, 13.31	<0.001	69	62	6.03	2.80, 12.98	<0.001
5	55	52	11.02	5.77, 21.05	<0.001	55	52	12.65	6.49, 24.66	<0.001

^a^ AFP, Alpha-Fetoprotein; ALBI, Albumin-Bilirubin; ALT, Alanine Aminotransferase; AST, Aspartate Aminotransferase; BCLC, Barcelona Clinic Liver Cancer; CI, Confidence Interval; ECOG PS, Eastern Cooperative Oncology Group Performance Status; HR, Hazard Ratio, PFS, Progression-free Survival; PLT, Platelet Count; PT, Prothrombin Time.

**Table 3:** Univariate and multivariate analysis of influencing factors for OS (Cox regression)

Characteristic	Univariable					Multivariable				
	*N*	Event *N*	HR^a^	95% CI^a^	*P*-value	*N*	Event *N*	HR^a^	95% CI^a^	*P*-value
ECOG										
0	318	187	—	—		318	187	—	—	
1	110	61	1.10	0.83, 1.47	0.504	110	61	0.85	0.63, 1.16	0.317
BCLC										
B	158	71	—	—		158	71	—	—	
C	270	177	2.11	1.60, 2.78	<0.001	270	177	1.80	1.08, 3.00	0.024
Vascular invasion										
Absent	214	106	—	—		214	106	—	—	
Present	214	142	1.70	1.32, 2.19	<0.001	214	142	0.70	0.46, 1.08	0.104
Tumor number										
≤3	174	99	—	—		174	99	—	—	
>3	254	149	1.18	0.92, 1.52	0.198	254	149	1.20	0.91, 1.58	0.203
Largest tumor										
≤10	275	140	—	—		275	140	—	—	
>10	153	108	2.12	1.65, 2.74	<0.001	153	108	1.20	0.88, 1.63	0.252
Metastasis										
Absent	277	146	—	—		277	146	—	—	
Present	151	102	1.82	1.41, 2.34	<0.001	151	102	1.04	0.74, 1.46	0.802
Imaging cirrhosis										
Absent	213	120	—	—		213	120	—	—	
Present	215	128	1.03	0.80, 1.32	0.844	215	128	1.08	0.81, 1.43	0.606
ALT										
≤40	221	123	—	—		221	123	—	—	
>40	207	125	1.21	0.94, 1.55	0.137	207	125	0.78	0.58, 1.06	0.119
AST										
≤40	154	76	—	—		154	76	—	—	
>40	274	172	2.08	1.58, 2.73	<0.001	274	172	1.70	1.17, 2.49	0.006
ALBI										
1	134	71	—	—		134	71	—	—	
2 or 3	294	177	1.22	0.92, 1.61	0.161	294	177	0.88	0.63, 1.24	0.466
AFP										
≤400 ng/ml	228	127	—	—		228	127	—	—	
>400 ng/ml	200	121	1.37	1.07, 1.76	0.013	200	121	0.68	0.42, 1.11	0.122
PLT										
>100	376	216	—	—		376	216	—	—	
≤100	52	32	1.08	0.75, 1.57	0.675	52	32	1.11	0.71, 1.72	0.644
Class										
2	28	5	—	—		28	5	—	—	
1	75	49	8.16	3.23, 20.60	<0.001	75	49	8.14	3.19, 20.80	<0.001
3	154	51	2.16	0.86, 5.41	0.102	154	51	1.82	0.65, 5.05	0.252
4	47	29	8.98	3.45, 23.41	<0.001	47	29	7.46	2.76, 20.19	<0.001
5	55	48	43.51	16.86, 112.26	<0.001	55	48	45.28	17.23, 119.03	<0.001
6	69	66	27.30	10.78, 69.11	<0.001	69	66	24.17	8.43, 69.27	<0.001

^a^ AFP, Alpha-Fetoprotein; ALBI, Albumin-Bilirubin; ALT, Alanine Aminotransferase; AST, AST; BCLC, Barcelona Clinic Liver Cancer; CI, Confidence Interval; ECOG PS, Eastern Cooperative Oncology Group Performance Status; HR, Hazard Ratio; OS, Overall Survival; PLT, Platelet Count; PT, Prothrombin Time.

## Discussion

The first finding showed no significant difference in median OS and PFS between the TACE-HAIC-MI and TACE-MI groups, both of which had acceptable safety profiles. Similarly, ORR and DCR were comparable between the two groups. After IPTW, the mOS was 23.9 months in the TACE-HAIC-MI group and 23.7 months in the TACE-MI group (*P* = 0.432); the mPFS in the two groups was 9.77 months and 9.77 months, respectively (*P* = 0.45). Although the TACE-HAIC-MI regimen appeared to reduce the risk of death in patients with BCLC stage C disease and extrahepatic metastasis, the difference between groups was not statistically significant.

The combination of TACE with HAIC, MTTs, and ICIs offers a complex treatment strategy targeting tumor growth through various mechanisms, potentially benefiting specific patients^[23]^. One study reported that HAIC increased surgical conversion rates in HCC patients with solitary tumors larger than 10 cm^[4]^. Some studies have suggested that HAIC combined with systemic therapy enhances efficacy in patients with HCC and portal vein tumor thrombus (PVTT)^[24–26]^. However, the superiority of HAIC over TACE with targeted immunotherapy remains debatable. Quadruple therapy with TACE-HAIC-MTTs-ICIs is still in the early stages of development. Pang *et al* reported a median OS of 18.2 months and PFS of 9.2 months for TACE-HAIC combined with tyrosine kinase inhibitors (TKI) and PD-1 inhibitors, although survival benefits appear limited compared to studies like CHANCE001 (median OS of 19.2 months for TACE-MI) and CHANCE2201 (median OS of 22.6 months for TACE-MI). Caution is advised when interpreting the conclusions from different retrospective studies. A multicenter study by Huang *et al* found that combining TACE-HAIC with atezolizumab and bevacizumab resulted in a median PFS of 10.1 months and an ORR of 62.2% in patients with high-tumor-burden HCC^[27]^. Yuan *et al* reported that TACE-HAIC with targeted and immune therapies outperformed TACE alone in patients with PVTT, with a median PFS of 14.8 months and a median OS not yet reached^[28].^ Additionally, Huang *et al* noted a survival benefit with TACE-HAIC-TKI-PD-1 over TACE-TKI-PD-1 in patients with unresectable HCC (21 vs. 14 months, *P* = 0.039)^[9]^. Notably, quadruple therapy had an acceptable safety profile in the above research, consistent with our study. Our research indicated that rates of nausea and vomiting were significantly higher (all *P* < 0.05), likely attributed to HAIC-related chemotherapy agents. Triple therapy was linked to increased liver function impairment (e.g., ALT/AST elevation), possibly from repeated TACE procedures.

The aforementioned studies indicate that the TACE-HAIC-MTTs-ICIs regimen may demonstrate greater efficacy compared to monotherapy approaches, with a median OS ranging from 10 to 21 months. However, there is a lack of comparative research between TACE-HAIC-MI and TACE-MI. Our study filled this gap and found no significant survival differences between the two treatments in patients with unresectable HCC. This challenges the assumption that a more complex regime yields better outcomes.

Notably, this study is the first to examine dynamic AFP changes in patients treated with TACE ± HAIC-MI and the results showed that changes are closely associated with survival outcomes. Patients with good responses with respect to AFP changes had significantly better survival outcomes than those with poor responses. Specifically, the most representative group was class 5 [low baseline AFP level (<100 ng/ml) with a gradual increase after treatment] which had the shortest median OS. In the multivariate regression analysis, compared to class 2 (persistent low-level group), the HR for class 5 was 45.28 (95% CI: 17.23–119.03), indicating a 45-fold increase in the risk of death. Even when compared to class 6, where baseline AFP was >10 000 ng/ml but showed a brief and slight decrease after treatment, class 5 had no survival benefit.

In this study, JLCM was used which included survival time and accounted for varying follow-up durations and AFP monitoring intervals by incorporating a quadratic polynomial term. The JLCM is distinguished from the latent class growth mixed model (LCGMM), generalized additive model (GAM), and latent class model (LCM) by synchronously integrating longitudinal dynamic trajectories and time-to-event data. This allows the identification of heterogeneous subgroup “trajectory-risk” association patterns through latent classes. A study of treatment with monotherapy of Lenvatinib reported that an early AFP response, defined as a reduction in serum AFP levels of more than 20% from baseline after 4 weeks of treatment, was associated with a higher ORR (34.5% vs. 6.3%), DCR (82.8% vs. 50.0%), and longer median PFS (13.0 months vs. 7.0 months) compared to nonresponders. Chao *et al* used the LCGMM model and identified three AFP trajectory patterns at various times in patients initially treated with HAIC. The results showed that HCC patients in the low-stability group had higher OS rates than those in the high-stability group (5-year OS rate: 22.7% vs. 7.6%; HR, 0.17; 95% CI, 0.13–0.24; p < 0.001) but lower OS rates than those in the sharp-falling group (5-year OS rate: 54.7%)^[29]^. Lu *et al* also identified three trajectories of AFP with LCGMM in HCC patients treated with TACE. Compared with the low-stable trajectory, the high-increase trajectory had a higher mortality risk [adjusted HR (aHR) = 5.13], while the sharp-decrease trajectory had a lower mortality risk (aHR = 0.52)^[30]^. A GAM was used to identify three distinct trajectories of AFP change in another study, and the results showed that patients in the sharp-falling group had longer OS than those in the low-stable and high-rising group. The results of a study using the LCM model to identify three AFP trajectories suggested that rapid reduction of AFP post-treatment can lead to favorable prognoses in patients treated with bevacizumab plus immunotherapy^[18]^. In summary, the current evidence demonstrates that a favorable AFP response is significantly associated with improved outcomes in HCC patients, a finding that aligns with our longitudinal trajectory analysis and highlights the prognostic value of dynamic AFP monitoring^[31,32]^.

In contrast to prior research, which predominantly classified dynamic changes in AFP into three patterns – stable, rising, and rapid decline, our model identified six distinct AFP trajectories, offering a more nuanced reflection of real-world data. Specifically, Figure 3 illustrates the stratification of patients into three baseline AFP levels: low (<100 ng/mL; log_10_AFP <2), intermediate (100–10 000 ng/mL; log_10_AFP 2–4), and high (>10 000 ng/mL; log_10_AFP >4). Following treatment, patients were further categorized based on AFP trajectories into groups characterized by either continuous elevation or gradual decline. Our analysis revealed that class 2, characterized by low baseline AFP with sustained low levels, demonstrated the most significant OS benefit. Importantly, class 3, which had high baseline AFP with subsequent decline to low levels, exhibited survival outcomes comparable to class 2. Conversely, class 5, defined by low baseline AFP with a continuous rise after treatment, was associated with the poorest prognosis. These results underscore the potential for individualized clinical guidance through the integration of baseline AFP levels and their dynamic changes. To assess the predictive stability of AFP trajectories, we conducted 2000 bootstrap internal cross-validations, resulting in a C-index of 0.787 (95% CI: 0.771–0.787) for survival prediction. This performance surpassed that of established multiparameter HCC prognostic models, including mHAP III (C-index(95% CI): 0.649 (0.610–0.688)), CRAFITY (C-index:0.62), Six-and-twelve (C-index(95%CI):0.66(0.63-0.69), and TACE-based nomograms (C-index:0.7555). Remarkably, our single-biomarker dynamic model demonstrated superior predictive accuracy compared to these conventional multimarker models.

In summary, our study addresses two critical knowledge gaps: first, by characterizing longitudinal AFP trajectories in patients receiving TACE with/without HAIC combined with MTTs and ICIs – a population underrepresented in existing literature; second, by advancing beyond conventional trajectory identification methods (including LCGMM, GAM, and LCM) that typically delineate only three AFP patterns and outperforming than base AFP value, common cutoff value for AFP in baseline level, and early AFP response^[15–17,33,34]^. This refined classification system enables clinicians to implement more personalized prognostic stratification and precision treatment strategies.

There are several limitations of this study that should be considered: (1) This was a retrospective study. The sample size was limited and there was also a lack of external validation. However, the multicenter nature of the data partially mitigates the limitations of this study. Besides, the model demonstrated robust stability upon 2000 bootstrap validation. However, prospective multicenter validation is warranted in the future. (2) The upper limits of AFP values varied between centers due to differences in AFP measurement instruments. This might have introduced minor differences in the AFP trajectory categories. However, trajectory analysis identified class 6 [patients with high baseline AFP (>10 000 ng/mL) and poor AFP response] as the subgroup with the worst prognosis, characterized by persistently elevated AFP levels (>10 000 ng/mL) during follow-up (Figure.S3. http://links.lww.com/JS9/E459). Notably, despite variability in detection limits, all instruments exceeded the critical threshold of 10 000 ng/ml observed in this high-risk subgroup, thereby minimizing the impact of upper-limit heterogeneity on trajectory classification and risk stratification to some extent. Furthermore, our findings align with the CHANCE2201 trial data, where TACE combined with targeted and immunotherapy demonstrated survival benefits in patients with baseline AFP >400 ng/ml. This supports the hypothesis that higher AFP thresholds may better stratify risks in patients receiving combination therapy. To validate this, we performed subgroup analyses using AFP >10 000 ng/mL as a novel cutoff and observed consistent prognostic discrimination (HR = 1.771, 95%CI:1.357-2.31) (Supplementary FigureS3. http://links.lww.com/JS9/E459), reinforcing the robustness of AFP thresholds. (3) Owing to the limited overall sample size, although the proportion of each category exceeded 5% after latent class analysis, the number of individuals in some categories was still small. This suggests the need for larger cohorts to further validate different AFP trajectories. 4) In the efficacy analysis of TACE-MI and TACE-HAIC-MI, baseline differences existed between the two groups. However, IPTW was used to reduce sample loss and balance baseline differences. Additionally, we performed sensitivity analyses using different weighting formulas to minimize potential biases from a single weighting method.

## Conclusions

First, unlike previous studies, the quadruplet therapy (TACE-HAIC-MI) showed no significant efficacy advantage. That challenged the notion that combination therapy is inherently superior. This underscores the need for greater individualization and precision when clinicians design combination regimens.

Second, given comparable efficacy, differing adverse reaction profiles offer treatment alternatives. Patients with poor gastrointestinal tolerance may favor TACE-MI, while those with severe liver impairment can potentially reduce TACE sessions (and thus liver injury risk) using TACE-HAIC-MI combination therapy.

Third, moving beyond the previous focus solely on high baseline AFP, trajectory analysis reveals that patients with high baseline AFP who achieve a good AFP response post-treatment still gain significant survival benefits. This highlights the importance of comprehensive management and continuous AFP monitoring, enabling clinicians to make timely follow-up treatment decisions based on AFP trends.

## Data Availability

The information generated or analyzed in this investigation can be obtained from the corresponding author upon reasonable request. This manuscript has not been published elsewhere and is not under consideration by another journal.

## Research registration unique identifying number (UIN)

Details omitted for double-anonymized peer review.
